# Reliable Adaptive Video Streaming Driven by Perceptual Semantics for Situational Awareness

**DOI:** 10.1155/2015/394956

**Published:** 2015-07-01

**Authors:** M. A. Pimentel-Niño, Paresh Saxena, M. A. Vazquez-Castro

**Affiliations:** Department of Telecommunications and Systems Engineering, Autonomous University of Barcelona, 08193 Bellaterra, Spain

## Abstract

A novel cross-layer optimized video adaptation driven by perceptual semantics is presented. The design target is streamed live video to enhance situational awareness in challenging communications conditions. Conventional solutions for recreational applications are inadequate and novel quality of experience (QoE) framework is proposed which allows fully controlled adaptation and enables perceptual semantic feedback. The framework relies on temporal/spatial abstraction for video applications serving beyond recreational purposes. An underlying cross-layer optimization technique takes into account feedback on network congestion (time) and erasures (space) to best distribute available (scarce) bandwidth. Systematic random linear network coding (SRNC) adds reliability while preserving perceptual semantics. Objective metrics of the perceptual features in QoE show homogeneous high performance when using the proposed scheme. Finally, the proposed scheme is in line with content-aware trends, by complying with information-centric-networking philosophy and architecture.

## 1. Introduction

Video services have become part of everyday interactions and contribute to a major portion of network traffic. Usage of video can surpass the recreational arena to provide additional insights in out-of-the-ordinary scenarios, such as emergencies or e-health aid.

Our interest is the use of live, point-to-point, beyond recreational video streaming. The goal of such service is to provide valuable information through the live video, in order to enhance the end-user's awareness of ongoing situations. We consider scenarios where best effort satellite networks become a reliable alternative to unavailable terrestrial communications infrastructure. However, these networks offer stringent conditions that challenge the user's satisfaction. The relevance of this realistic scenario is supported by direct contact and fieldwork with decision makers using these types of video services during disaster and emergency response events [1].

In this paper, we propose a novel, complete model and solution for live video transmission of user generated to enhance situational awareness. It is composed of novel QoE framework that allows fully controlled adaptation and enables perceptual semantic feedback. The framework is based on temporal/spatial abstraction for video applications serving beyond recreational purposes. To the best of our knowledge, the aim of offering complete solution for the given scenario requirements (video for beyond recreational needs to enhance awareness) and its constraints (challenging communications with scarce bandwidth suffering from both congestion and erasures) has not been addressed before. Moreover, we use novel tools to tackle these issues and provide robust and feasible scheme.

*Existing Strategies for Congestion Avoidance*. Video streaming is growing in popularity on the best effort internet. Existing technologies such as dynamic adaptive streaming over HTTP (DASH), layered on top of TCP, have well-known advantages. However, they are not applicable in our design for situation awareness where, for example, satcom scenarios need to be considered. In such scenarios, TCP has low performance for streaming due to long round trip times and packet erasures. This results in a decrease in throughput that causes long start-up delays in video playback and bounds the video source rate to just half of the TCP throughput [2]. At the same time, the topology of our scenario is not compatible with HTTP/TCP—HTTP options of user generated live (close to real-time) streaming rely on intermediate servers to prepare content for the clients (e.g., in order to use DASH). All of the above affect QoE with freezes in playback and low image quality. As an alternative, improvements to general purpose TCP for satellite communications include performance enhancement proxy (PEP) solutions; however, they alter the system architecture.

A variety of TCP-friendly congestion control schemes have been studied for video streaming [3–5]. Their main focus is the fairness of the schemes rather than the impact on QoE. Further, they rely on heavy feedback from the receiver, which is a problematic issue in long-delayed networks. Real-time applications, on the other hand, opt for real-time protocol (RTP) and use ad hoc congestion control schemes [6, 7]. They offer the flexibility our scenario needs at the transport layer; however, they rarely focus on QoE assessment or on addressing band-limited long-delayed networks.

In this work, we propose congestion avoidance over RTP in conjunction with video adaptation, specifically for QoE in satellite networks. We use utility-based optimization approach, based on [8, 9]. Related work, as in [10, 11], is driven by QoS performance objectives. Other approaches use parameterizations from standardized quality of video to obtain video quality adaptive algorithms [12]. Finally, mappings of subjective QoE metrics are also used as optimization functions [13]. The drawback of the aforementioned approaches is the heterogeneity in the choices for mapping and scenarios, which may not be reproducible or generalized for broader adoption. Furthermore, the existing approaches do not address long roundtrip times, which render them unsuitable for our scenario.

*Network Coding for Improving Reliability*. In general, wireless systems (specifically satellite systems) suffer from packet erasures. These erasures are due to poor wireless reception conditions and channel fading among others, which the adaptive coding schemes at the physical layer cannot cope with. State-of-the-art video codecs include error concealment features for robustness against erasures; however, they only suffice for short temporal error propagation and may not handle more severe losses [14].

Existing erasure recovery strategies primarily include retransmissions or the use of redundant packets. Retransmission based schemes (e.g., ARQ and TCP) may provide perfect erasure recovery. However, the increase in delay and overhead due to per packet feedback can decrease throughput, especially when round trip times are high. Rateless coding schemes like Luby transform (LT) codes [15] and Raptor codes [16] can generate a fountain of redundant packets and are especially popular for reliable transmission of large files. However, these codes are not efficient with small block sizes, which is the usual structure in video streams [16].

In this work, we examine the use of block coding with random network coding (RNC) to improve reliability. RNC [17, 18] allows mixing of packets to send a fixed amount of redundant packets such that there is sufficient protection guaranteed and the source packets are recovered without the need of feedback. It also provides the inherent possibility of reencoding at intermediate nodes (which is missing in the traditional block coding schemes). The use of RNC for reliable communications in wireless networks was first studied in [19] where RNC was shown to achieve maximum throughput for both unicast and multicast communication with packet erasures. In addition, [20–22] discuss the use of RNC for reliability.

In this paper, we focus on systematic random linear network coding (SRNC). SRNC provides an erasure recovery performance similar to maximum distance separable (MDS) codes such as Reed Solomon (RS) codes [23]. In addition, with systematic codes input data is embedded in the encoded output, thereby reducing decoding overhead at the receiver side. Furthermore, the inherent random structure of SRNC makes progressive decoding possible, which improves packet recovery time as compared to using RS codes. This is an advantage in long-delayed scenarios.

*QoE and Semantics*. QoE is a multidisciplinary field that aims to understand the degree of human satisfaction with an application or service. General QoE models for telecommunications integrate different aspects into a holistic view of QoE [24]. A thorough review of general purpose QoE models and QoE management for wireless networks can be found in [25].

With respect to QoE for video in particular, several features have been studied to improve user's experience in streaming, such as video coding parameters [26] or temporal impairments [27, 28]. Such solutions focus separately on erasure protection solutions for lossy networks or on dynamic rate adaptation for best effort cases [13, 29].

In contrast to the aforementioned approaches, we base the notions of QoE upon [30], focusing on (1) system influential factors on QoE that relate to the networking scenario at hand and (2) perceptual features in QoE for video that will guarantee delivery of valuable information. Moreover, we follow QoE versus QoS correlation modeling approaches [31], where our aim is high temporal (related to congestion avoidance) and spatial (related to reliable transmission) QoE procurement.

Finally, we propose an additional dimension to our framework that targets specific user demands for situational awareness. In multimedia, “classic” semantics deals with heterogeneous metadata that sensors observe and/or tag when capturing video. It has applications in information retrieval, integration, and aggregation of varied data types such as semantic-aware delivery of multimedia [32]. Furthermore, semantic tagging describing pure observations is used in computer-based systems with artificial intelligence to perceive and abstract situations [33]. Rather than doing perception through classic semantics, we propose a novel human-analysis-driven perceptual semantics approach to tag videos based on the spatial/temporal characteristics of the video a user is perceiving. This provides a mechanism to specifically target and improve the user's perceptual needs and enhance situational awareness.

*Contributions*. The main contributions of this paper can be summarized in terms of the following novel aspects:

- Novel scenario: we concentrate on the use of video to fulfill beyond recreational needs, for example, situational awareness in critical situations; hence the key issue is the iterative use of video over scarce bandwidth. Under these circumstances, video transmission cannot be thought of as a standard streaming solution for domestic use over the internet, nor can it rely on well-known and available encoders or solutions, but on very robust and well-controlled adaptation and coding.
- Novel decoupling of time/space for the video adaptation/coding: we address the user's specific perceptual demands and map, in time and in space, the corresponding network triggers that degrade the user's perceptual awareness. Based on this mapping, we propose a robust and controlled optimization by decoupling the time and space domains. In addition, this approach proves to be useful in tackling systematically the stringent restrictions of our communications scenario and meets the user's demands.
- Novel perceptual semantic level: we propose a novel perceptual semantics dimension that is intrinsically related to the situational awareness scenario and the end-user driven nature of our approach. Such problems have not been addressed by current state-of-the-art video streaming solutions that focus mainly on communication for recreational needs. The end-user is involved in interactive adaptation through perceptual semantics feedback such that specific user-demanded perceptual spatiotemporal enhancements are possible. Furthermore, it is compliant to current content-aware networking trends.
- The use of network coding: network coding is used to improve reliability. It proves to perform similar to MDS codes, with several advantages over MDS codes including reduced decoding complexity, smaller delay, and flexibility to perform adaptive coding. In addition, this scheme allows the extension of coding at intermediate nodes in more complex networking scenarios, providing better performance in terms of throughput and reliability.
- Experimental validation: we show through a time/space graphical analysis that the joint optimizations achieve planar, homogeneous performance with high values of QoE metrics. This performance is achieved regardless of both erasures and congestion degrading the network. In addition, both optimizations guarantee good performance of the perceptual semantics level to meet the user's perceptual demands for situational awareness. Moreover, our framework has proven to be of high relevance in realistic scenarios [1].

The rest of the paper is organized as follows. In Section 2 we present the scenario. In Section 3, we discuss the system model. In Section 4, we present the QoE optimization in the time domain.  Section 5 discusses the QoE optimization in the space domain.  Section 6 presents the integration of perceptual semantics into the framework. In Section 7, we present our experimental results. Finally, we present concluding remarks in Section 8.

*Notation*. Let *𝔽*_*q*_ be a finite field. We denote *𝔽*_*q*_^*a*_1_×*a*_2_^ as the set of all *a*_1_ × *a*_2_ matrices with entries in *𝔽*_*q*_ and *𝔽*_*q*_^*a*_1_^ as the set of all column vectors with *a*_1_ entries in *𝔽*_*q*_. Boldface uppercase letters are used to denote matrices and boldface letters to denote column vectors. **I**_*a*_ is used to denote *a* × *a* identity matrix. The notation ∪**I**_*a*_^*a* × *a*_1_^ represents the set that contains *a*_1_ distinct columns of identity matrix **I**_*a*_. ∇_*R*^·^_ denotes the gradient with respect to *R*.

## 2. Scenario

We consider point-to-point live streaming of user generated video content for beyond recreational purposes in challenging communications scenarios. The end-user is receiving the live stream and has the possibility of demanding enhancements of video features interactively. The received stream is helping the user improve his/her awareness of the situations depicted in the video, with no use of artificial intelligence in the perceptual and awareness processes [34, 35]. Emergencies, monitoring, or telemedicine are an example of potential scenarios.

### 2.1. Spatiotemporal Abstraction of Video Services Beyond Recreational

We propose spatiotemporal abstraction that is closer to the perceptual demands of the user. This abstraction is inspired by situational awareness scenarios [36] and diverges from traditional spatiotemporal concepts in video coding, for instance.

The spatial abstraction refers to precise time-space accounts of an ongoing situation such as precision of details and accuracy for identification in a crowd. The temporal abstraction refers to insights in the temporal aspects of dynamically changing situations such as evolution of events and temporal tracking [34].

### 2.2. Networks in Emergency Scenarios

We assume a sender with access to a band-limited communications network. We consider portable/mobile IP-based satellite services, such as the broadband global area network (BGAN) [37], often used in emergency scenarios, provided by a network of geostationary satellites. These types of services, while ubiquitous, offer limited broadband capacity compared to state-of-the-art wireless terrestrial mobile networks. In addition, inherent long propagation delays are present (in particular in geostationary satellite topologies) and losses from the wireless medium render the network unreliable. As a generalized case we consider best effort provisioning since guaranteed services may not be available. Congestion is thus present.

## 3. System Model

### 3.1. QoS/QoE Modeling by Time/Space Decoupling

#### 3.1.1. System Influential Factors Indicators

**Definition 1 .**

*Quality of Service, QoS*, is the ability of the network or service to provide or guarantee a certain level of performance for a data flow.

We consider the following QoS metrics to quantify the influence of the effects of congestion and erasures in the best effort satellite scenario.

**Definition 2 .**

*Erasure rate ϵ* is a random variable that follows an i.i.d random process. It represents packet erasure rate due to channel fading in wireless links.

**Definition 3 .**

*Congestion-induced erasure rate ϵ*_*c*_ is a random variable that follows an i.i.d random process. It represents packet erasure rate due to congestion in best effort wireless networks.

**Definition 4 .**

*Network delay τ* is used as an indicator of congestion.

**Definition 5 .**

*Degree of congestion η* represents how congested the network is with respect to the maximum available rate offered *r*_av_^max^.  *η* = *r*_av_/*r*_av_^max^, where 0 < *η* ≤ 1 and *r*_av_ ≤ *r*_av_^max^ is the available rate to the user at any given time. A value of *η* tending to 0 indicates severe congestion, while *η* → 1 indicates no congestion. (*r*_av_^max^ depends on the underlying network (i.e., for the BGAN network in the best effort mode *r*_av_^max^ ≈ 500 kbps)).

#### 3.1.2. QoE Framework

We first present the framework used to decompose the system and perceptual aspects of the scenario in Section 2, according to standard QoE definitions.

**Definition 6 .**

*Quality of experience, QoE*, is the degree of delight or annoyance of the user of an application or service [30]. Continuing with the taxonomy proposed by [30], QoE is decomposed into influential factors and perceptual features.

*(a) QoE System Influential Factors*. These factors signify the technical aspects affecting quality of the application or service, such as media capture, coding, transmission, and playback. Such factors may lead to noticeable degradations such as artifacts, blockiness, and freezes. In the scenario considered in this paper, the QoE system influential factors are linked to the underlying network performance, for example, the best effort wireless satellite network and its QoS.

Other influential factors affecting QoE are context and human factors. Human factors, surrounding emotional and sociological backgrounds, are out of scope of this paper. Context aspects can be a natural extension of the work we present here.

*(b) Perceptual Features of QoE*. These features are the perceivable characteristics of a user's experience contributing to the overall quality [30]. These features are directly linked to our spatiotemporal abstraction for video services presented in Section 2. Henceforth, we distinguish a time and space domain decomposition based on the spatiotemporal perceptual features in QoE.

In the space domain, we refer to user's dissatisfaction due to a lack in accuracy, artifacts in the video caused by packet erasures, and source coding distortion, among others. In the time domain, we refer to user's dissatisfaction due to persistent freezes in the video playback that prevent tracking dynamically changing situations. In Section 7.2 we discuss the metrics used to measure the spatiotemporal perceptual features of QoE.

#### 3.1.3. QoS/QoE Mapping

**Figure 1:**
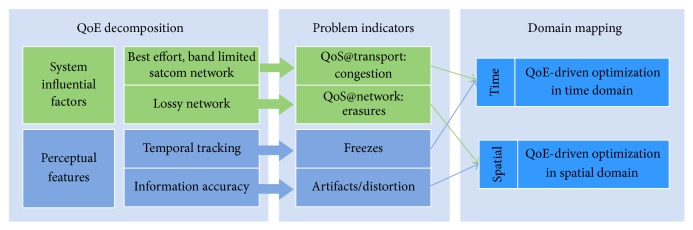
Scenario-specific QoE framework with decoupling in time and space domains of QoE.

Figure 1 summarizes the proposed time-space decomposition. Our analysis is as follows.

Congestion affects QoE primarily in the time domain, inducing freezes in video playback. If congestion can be tracked at the transport layer, rate adaptation to the network's available rate can be performed and QoE in the time domain will be improved.

Erasures affect QoE in the space domain, inducing artifacts in video. Channel coding in the network layer can help recover from erasures, thereby improving QoE in the space domain.

By mapping congestion to the time domain and erasures to the space domain, we are able to propose decoupled solution for the joint problem affecting our scenario. Hence, we propose two QoE-driven optimizations, jointly operative but working separately, one for the time domain and the other for the space domain, to work at the transport and network layers, respectively.

A decoupled solution provides advantages in terms of flexibility of the design since the formulation and performance evaluation of the two optimizations can be treated separately. A potential concern is whether the optimizations can affect one another when they are operating at the same time. In Section 7 we show that, under reasonable assumptions, this cross-influence is minimal.

### 3.2. Perceptual Semantics Model

Classic semantics based approaches typically use unprocessed sensorial observations [33]. Our proposed perceptual semantics approach represents more complex abstractions of a viewed scene. Based on the spatial/temporal abstraction for video services in Section 2 and its mapping to perceptual features in QoE as shown in Figure 1, our proposal is to utilize the end-user's (analyst) perception, to do semantic tagging that enables an enhancement of the received video stream signal tailored to the user's demand.

We propose perceptual tagging that indicates the spatial/temporal predominance according to the level of perception of the user. A tag indicating predominance of temporal features implies that the user is perceiving a situation that demands more attention to the dynamics of the scene (e.g., rapid movements). On the other hand, predominance of spatial features indicates moments of less movement but densely overloaded frames, which requires more detail to identify features.

In scenarios where perception is not achieved by artificial intelligence, human analysis interprets the sensory information (i.e., perceiving). Hence, we propose semantic tagging to be performed by the user, as he/she is ultimately the one perceiving and foreseeing what might be of interest in the video.

### 3.3. Topology

**Figure 2:**
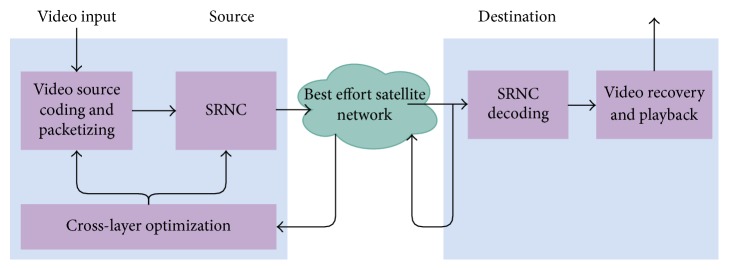
Scenario.

We consider a point-to-point scenario where the underlying satellite network topology can have several intermediate nodes. In this paper we consider channel coding for reliability only at the source node. However, our system model can be extended to allow network coding at intermediate nodes of the network (this is out of the scope of this paper and a part of ongoing work).  Figure 2 shows the overall block diagram of the source-destination topology and our proposed solution.

### 3.4. Cross-Layer Optimization

As seen in Figure 1, we propose a decoupled cross-layer QoE-driven optimization framework consisting of two types of optimization, one in the time domain and the other in the space domain of QoE.

**Figure 3:**
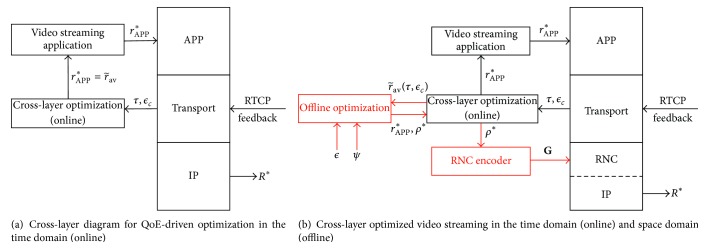
Proposed cross-layer optimization framework.

In the time domain, as shown in Figure 3(a), we use an online adaptation strategy that uses end-to-end feedback at transport layer to cope with congestion. Network delay *τ* and congestion-induced erasures *ϵ*_*c*_ can be inferred from the feedback and used to estimate ${\tilde{r}}_{av}$. The application layer rate *r*_APP_^*∗*^ to be used by the video streaming application is $r_{APP}^{*}={\tilde{r}}_{av}$. As a result, the transmission rate at the network layer *R* is *R* ^*∗*^ ≈ *r*_APP_^*∗*^(we consider overhead due to layer encapsulation to be negligible when calculating the rates) after optimization, which matches the application layer rate.

Figure 3(b) shows the integration of the QoE optimizations in the time and space domains. The available rate ${\tilde{r}}_{av}$ is first estimated online by the optimization in the time domain. ${\tilde{r}}_{av}$ is used as input to the optimization in the space domain to obtain the optimal code rate *ρ* ^*∗*^ for erasure protection using RNC coding. As a result, the application layer rate is adapted to $r_{APP}^{*}=\rho^{*}{\tilde{r}}_{av}$ and the transport layer packets are encoded using RNC at a sublayer of the network layer. Finally, the IP sublayer transmits at a rate $R^{*}\approx {\tilde{r}}_{av}$. The optimization in the space domain can be performed offline, and look-up tables can be available online with optimal values for a certain set of input values.

The online adaptation strategy, resulting in dynamic rate adaptation, fulfills two purposes, namely, (1) congestion control at transport layer and (2) online adaptation of the video source for maximized QoE.

Note that RNC is chosen to be at the network layer in order to enable the possibility of coding at intermediate nodes in future work. Network layer packets are accessible at intermediate nodes; hence coding at this layer would be more efficient in our model.

### 3.5. Matricial System Model

We consider the frame structure of standard state-of-the-art video codecs to model the source. Coded frames are grouped into groups of pictures (GoPs). Each GoP has three types of frames, namely I, P, and B frames, each of different importance. Network coding can be used to provide unequal protection (UEP) for these different frames [9]; however, in this work, we consider equal protection and focus on the allocation of redundant packets to the complete GoP block.

We consider each GoP to have a fixed number of frames *N*_frame_. The frame rate *r*_fr_ is such that the codec outputs each GoP in a fixed time *T*_GoP_ = *N*_frame_/*r*_fr_. We denote *N*_GoP_ as the total number of GoP's output by the codec during the entire streaming session such that *N*_GoP_ × *T*_GoP_ = *T*.

The codec outputs the *n*th GoP, coded at application layer rate *r*_APP_, for *n* ∈ {1,2,…, *N*_GoP_} at time *t*_*n*_ ∈ {0, *T*_GoP_, 2*T*_GoP_,…, (*N*_GoP_  −  1)*T*_GoP_}. Although *N*_frame_ is fixed, frame sizes vary depending on the *r*_APP_. For the *n*th GoP, each frame is fragmented into multiple packets of equal length *l* (in bits) and delivered from the transport layer to the (RNC + IP) layer for end-to-end delivery. We denote *K*(*n*) = ⌈(*r*_APP_ × *T*_GoP_)/*l*⌉ as the total number of packets from the *n*th GoP. We drop the index *n* for simplicity in formulation; however, as the source *r*_APP_ is time varying, coding parameters depending on *r*_APP_ can also vary from one GoP to another.

We define **S** ∈ *𝔽*_*q*_^*M* × *K*^ as containing all the packets from a GoP. Each packet is a column vector of *M* symbols where *M* is a function of field size *q* with packet length *l*, given by *M* = *l*/log_*ω*_⁡*q*, and *ω* is a prime number called the characteristic of the field.

Encoding is done at the RNC layer as shown in Figure 3(b). The encoding process is linear such that the *N* coded packets corresponding to *K* source packets are given by

(1) $$\begin{array}{c} X=SG, \end{array}$$

where **X** ∈ *𝔽*_*q*_^*M* × *N*^. **G** ∈ *𝔽*_*q*_^*K* × *N*^ is the corresponding generator matrix of linear (*N*, *K*) code, with *N* = *K*/*ρ*, where $\rho =r_{APP}/{\tilde{r}}_{av}$ is the code rate used for encoding.

Specifically in SRNC, the *N* coded packets are composed of the embedded *K* source packets (systematic phase) and the redundant *N* − *K* packets, product of linear combination of the source packets (nonsystematic phase). Hence, the generator matrix *G* results in **G** = [**I**_*K*_ **C**], where **C** ∈ *𝔽*_*q*_^*K* × *N* − *K*^ is a matrix with random coefficients from the finite field *𝔽*_*q*_. The linear combining is random and it is not constrained to specific combination of coding parameters. This allows us to have flexibility in choosing coding parameters (*N*, *K*) which may vary from one GoP to another.

After the addition of IP headers, these *N* IP packets are transmitted to the destination over an erasure channel where the packets can be erased. We denote the channel function *ℋ* : *𝔽*_*q*_^*M* × *N*^ → *𝔽*_*q*_^*M* × *L*^, which maps *N* encoded packets to *L* received packets. We denote the received unit by the matrix **Y** ∈ *𝔽*_*q*_^*M* × *L*^ such that each received packet is a column vector of *M* symbols. In our case, the channel model is linear and we have **Y** = **X** **H** = **S** **G** **H**, with **H** ∈ ∪**I**_*N*_^*N* × *L*^.

The matrix **H** represents the erasure of packets, consisting of all the columns of **I**_*N*_ except the columns *i* ∈ {1,2, ,…, *N*} if the *i*th column/packet is erased by the channel. The channel matrix deletes the packets of **X** which are lost and hence **Y** consists only of received packets.

## 4. Optimization in the Time Domain

In this section we present the formulation and implementation of the QoE-driven optimization in the time domain, as part of the cross-layer model in Figure 3. In our QoE decoupling approach, we have identified congestion with freezes in the video playback and hence propose an optimization for improved QoE in the time domain. The implementation of this optimization results in a dynamic rate adaptation algorithm.

### 4.1. Formulation

Consider a best effort wireless scenario with a network varying over time *t*.

The general formulation of our objective optimization problem is presented in (2), where the utility function *U* is dependent on the QoS parameters. The QoS parameters considered are the transmission rate *R*, delay *τ*, and congestion-induced erasures *ϵ*_*c*_, all of them varying with time *t* (*R*, *τ*, and *ϵ*_*c*_ are function of time *t*; for clarity in stating the optimization problem we have dropped *t* in (2)–(5)).  *r*_av_^max^ is the upper bound on maximum rate offered by the network. The available rate *r*_av_ ≤ *r*_av_^max^ offered by the network may vary over time and is unknown to the user. Consider

(2) R∗=argmaxR UR,τ,ϵc,s.t. R≤ravmax.

Consider an additive model, where the utility *U* is composed of two functions, namely, one representing QoE's improvement with increasing assignment of network resources and a second one representing the dynamics degrading the network in the best effort scenario. Consider

(3) $$\begin{array}{c} U\left(\;R,\tau ,\epsilon_{c}\;\right)=U_{QoE}\left(\;R\;\right)-U_{QoS}\left(\;R,\tau ,\epsilon_{c}\;\right), \end{array}$$

where *U*_QoE_(*R*) is a concave function, defined in (4) based on the logarithmic mappings from QoS to QoE. Studies have shown that if the rate is increased in a controllable fashion (e.g., by increasing the application layer rate of the video), QoE behaves with a logarithmic relationship [39]. Consider

(4) $$\begin{array}{c} U_{QoE}\left(\;R\;\right)=\kappa \cdot \log\left(\;R\;\right),\;\kappa >0. \end{array}$$

*U*_QoS_(*R*, *τ*, *ϵ*_*c*_), on the other hand, expresses the penalizing effect of a congested network scenario, where injecting higher rate than the currently available one for the user (*r*_av_) translates into accumulating delay *τ* and eventually an overflow of network buffers leading to packet losses. Hence, we formulate *U*_QoS_ as a bilinear function of *τ* and *R* in

(5) $$\begin{array}{c} U_{QoS}\left(\;R,\tau ,\epsilon_{c}\;\right)=\gamma \left(\;\tau ,\epsilon_{c}\left(\;\tau \;\right)\;\right)\cdot \tau \cdot R. \end{array}$$

Notice that we define the function *γ*(·) > 0 to strengthen or weaken the effect of *U*_QoS_ in the overall optimization depending on the level of congestion perceived, as proposed in [40, 41], for flow control applications.

### 4.2. Implementation as Dynamic Rate Adaptation

#### 4.2.1. Solution to the Optimization Problem

**Proposition 7 .**

The optimization problem stated in (2) where the utility function *U* is defined as in (3) is solved using the discrete rate update algorithm (6), to find the value of *R* at time *t*_*k*+1_, for *k* ∈ *ℕ*, where *T*_*samp*_ = *t*_*k*+1_ − *t*_*k*_ is the network sampling time, *δ* is the step size, and ∇_*R*^·^_ is the gradient with respect to *R*. Consider

(6) $$\begin{array}{c} R\left(\;t_{k+1}\;\right)=R\left(\;t_{k}\;\right)+\delta \left[\;{\left.\;\nabla_{R}U_{QoE}\;\right|}_{t=t_{k}}-{\left.\;\nabla_{R}U_{QoS}\;\right|}_{t=t_{k}}\;\right]. \end{array}$$

**Proof**

First we prove that *U* is concave and hence an optimal value *R* ^*∗*^ that solves (2) exists. The function *U*_QoE_ from (4) is strictly concave increasing with *R*, while −*U*_QoS_ is concave, decreasing with *R*. The sum of concave functions is concave; hence *U*is concave and an optimal *R* ^*∗*^ ≤ *r*_av_^max^ that solves (2) exists. Further, the gradient ascent method can be used to find the optimal *R* ^*∗*^, where *R* is varying over time in the direction of the positive gradient of *U* : *dR*/*dt* = ∇_*R*_ *U*. In practice, rate updates happen in discrete time, and if we consider sampling time *T*_samp_ = *t*_*k*+1_ − *t*_*k*_, the rate control update is expressed as in (6).

Observe that ∇_*R*_ *U*_QoS_ changes with the current network conditions at time *t*; as a result, knowledge of QoS levels at the transport layer is needed to solve the optimization problem. Such knowledge of the network is based on feedback from the receiver end. If we consider feedback delay, the measurements represent QoS levels at delayed points. This is especially true in the case of long delay networks, such as satellite networks, where propagation delay is noticeable.

**Proposition 8 .**

In the case of a delayed network, with propagation delay *τ*_*D*_, the algorithm in (6) that solves (2), with *U* defined from (3), (4), and (5), is expressed as in

(7) Rtk+1=Rtk+δκRtk−γtk−τDτtk−τD.

**Proof**

The algorithm is triggered when new network measurements are available at the sender side; however, those are measurements corresponding to the network state at time *t*_*k*_ − *τ*_*D*_. Furthermore, if we consider the rate control update with sampling time to be greater than the network's roundtrip time (*T*_samp_ > *T*_RTT_), we can assume that the receiver is able to report on network changes related to the last rate control action from the sender at time *t*_*k*_. Consequently, we can express (6) as *R*(*t*_*k*+1_) = *R*(*t*_*k*_) + *δ*[∇_*R*_ *U*_QoE_|_*t*=*t*_*k*__ − ∇_*R*_ *U*_QoS_|_*t*=*t*_*k*_−*τ*_*D*__], where the gradient of *U*_QoE_ is evaluated at time *t*_*k*_ and the gradient of *U*_QoS_ is evaluated at time *t*_*k*_ − *τ*_*D*_. Substituting *U*_QoS_ and *U*_QoE_ for (5) and (4) we obtain (7).

The function *γ*(·) is chosen such that the response of the adaptation depends on the current measurements indicating congestion and can react faster to increasing delay constraints and packet loss as described by (8), where *t*_*i*_ = *t*_*k*_ − *τ*_*D*_. Note that *γ*(*t*_*i*_) responds to increases in *ϵ*_*c*_, which are accompanied with increases in delay *τ*. Hence other sources of packet erasures not related to congestion will not trigger a change in the rate control update. Consider

(8) γti=γti−1,γti−1=γt0,  τti≤τmax,λγti−1,τti>τmax,  ϵcti>ϵcmax,γti−1−1γti−1,γti−1>γt0,  τti≤τmax.

The delay-driven rate update obtained from (6) using the value of *γ*(·) according to (8) provides a smooth (an advantage to user's QoE [29]) output that is also capable of reacting fast to severe degradations in QoS. *γ*(*t*_0_) and *λ* > 1 are chosen for a desired response time, while *τ* ^max^ and *ϵ*_*c*_^max^ correspond to upper bound limits to *τ* and *ϵ*_*c*_, set according to application requirements.

#### 4.2.2. Cross-Layer Aspects and Practical Issues

The optimization proposed in this section is used within the whole cross-layer framework in Figures 3(b) and 3(a), such that $R^{*}\approx {\tilde{r}}_{av}$. Further, in order to maintain coherence of our model in time and avoid synchronization issues at different layers, we assume that *T*_GoP_ < *T*_samp_. The obtained application layer rate *r*_APP_^*∗*^ for the *n*th GoP is thus invariant for the duration of the whole GoP.

Cross-layer feedback from the receiver is provided by the real-time control protocol, RTCP, (RFC 3550) with a frequency of reporting of 1/*T*_samp_. In order to obtain *ϵ*_*c*_ and *τ* to estimate *R* ^*∗*^, the following fields from both sender and receiver RTCP reports are required (following RFC 3550 standard):* fraction lost*,* delay since last report (DLSR)*, and* last sent report (LSR)*.

The obtained algorithm results in high granularity rate adaptation. Therefore it requires a video codec capable of performing on-the-fly encoding with fine granularity. The standard codec H.264/AVC offers such features, with possibility of adaptation of its quantization parameters (QP) at encoding time. The VP8 codec also offers such capabilities [42], with the option of real-time encoding with on-the-fly reconfiguration of application layer rate. The extension of the H.264 codec for scalable video coding (SVC) could also be considered under certain assumptions. SVC would offer more coarse granularity in achieving the rate *R* ^*∗*^, depending on the combinations of temporal/spatial/amplitude scalability layers. Therefore, additional buffering might be needed in order to diminish potential impact on congestion. Further, computational complexity during real-time coding of all layers would increase.

## 5. Optimization in the Space Domain

In our decoupling approach, we identify erasures with artifacts in the video to be solved using an optimization in the space domain. In this section, we present the formulation and implementation of such QoE-driven optimization, as part of the cross-layer model in Figure 3.

The objective of the optimization in the space domain is to optimize application rate *r*_APP_^*∗*^ and code rate *ρ* ^*∗*^, in order to use SRNC to cope with erasures with maximized QoE of video.

### 5.1. Formulation

Let us consider ${\tilde{r}}_{av}$ to be the available rate estimated using the algorithm in (6). In order to protect the video stream from network erasures, SNRC coding will be used with a certain allocated code rate *ρ*. A low value of *ρ* implies more erasure protection, at the expense of a lower rate for the application layer $\left(r_{APP}=\rho {\tilde{r}}_{av}\right)$. Given that a lower *r*_APP_ results from higher compression rates, QoE in the space domain is damaged with low values of *ρ*.

Hence, we propose in (9) maximizing QoE by maximizing *r*_APP_, such that SRNC is used with an optimal code rate *ρ* ^*∗*^ that guarantees a residual erasure rate *ψ*. Consider

(9) rAPP∗=max⁡ rAPP,s.t.  rAPP≤r~av, ϵresϵ,q,rAPP,r~av≤ψ,

where $\epsilon^{res}(\epsilon ,q,r_{APP},{\tilde{r}}_{av})$ is the residual erasure rate of SRNC with field size *q*.

We target an offline solution to (9) in order to obtain the optimal values (*r*_APP_^*∗*^ and *ρ* ^*∗*^) corresponding to all the possible estimated available rates ${\tilde{r}}_{av}$. A look-up table with these values is generated. As ${\tilde{r}}_{av}$ is time varying and is estimated from feedback, the look-up table is accessed online and optimal *r*_APP_^*∗*^ and *ρ* ^*∗*^ are obtained corresponding to ${\tilde{r}}_{av}$. Consider
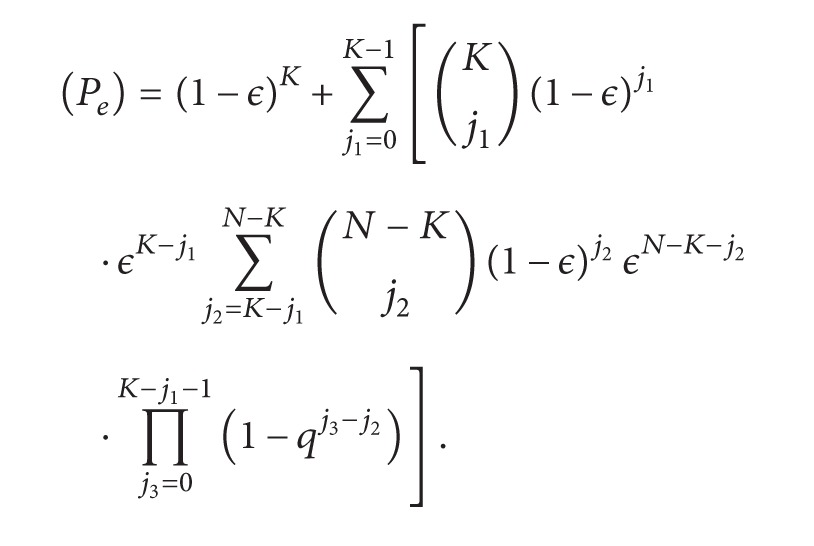
(10)


### 5.2. Implementation Based on SRNC

In this section, we present the implementation of SRNC and its performance.

Following the matricial model in Section 3, we have *K* = ⌈(*r*_APP_^*∗*^ × *T*_GoP_)/*l*⌉ and *N* = *K*/*ρ* ^*∗*^ depending on *r*_APP_ and ${\tilde{r}}_{av}$. Hence the residual erasure rate *ϵ* ^res^ is expressed as a function of *K* and *N* − *ϵ* ^res^(*ϵ*, *q*, *K*, *N*). Moreover, as noted earlier, we receive **Y** = **X** **H** = **S** **G** **H** consisting of *L* coded packets for each GoP. From the received unit **Y**, we can obtain the source unit **S**, when (i) **G** **H** is known at the receiver and (ii) rank⁡(**G** **H**) = *K*. If these two conditions are fulfilled, then at the destination source packets are obtained by **S** = **Y**(**G** **H**)^−1^.

In order to recover **G** **H** we use pseudorandom network coding [43]. The index of the codebook is sent along with each coded packet (the columns of **X**). The receiver, which has the same codebook, can recover **G** **H** with this index. The advantage of this option is low overhead (only the index per packet) compared with the overhead of sending the coefficients (each column of **G**) attached to each coded packet [17].

Gauss-Jordan elimination (instead of Gaussian elimination) is used for progressive decoding. As a result, it is not necessary to wait for the complete block and packet recovery time is reduced. In comparison, state-of-the-art RS codes require a complete block to start decoding.

The source packets are finally recovered if rank⁡(**G** **H**) = *K*. Let us denote *P*_*e*_ = *P*{rank⁡(**G** **H**) = *K*} as the probability of successfully decoding the original packets. Based on *P*_*e*_, we evaluate the residual erasure rate for SRNC in the next proposition.

**Proposition 9 .**

If SRNC is used with field size *q* and *K* source packets (from the systematic phase) with *N* − *K* redundant packets (from the nonsystematic phase) are transmitted over the erasure channel with random erasure rate, *ϵ*, then the residual erasure rate for SRNC is given by

(11) $$\begin{array}{c} \epsilon^{res}\left(\;\epsilon ,q,K,N\;\right)=1-{\left(\;P_{e}\;\right)}^{1/K}, \end{array}$$

where *P*_*e*_ = *P*{rank⁡(**G** **H**) = *K*} is given by (10).

**Proof**

We will first evaluate *P*_*e*_. Let *j*_1_ and *j*_2_ be the number of packets received from the systematic and nonsystematic phases, respectively. *j*_1_ ≤ *K*, *j*_2_ ≤ *N* − *K*, and the dimensions of **G** **H** are *K* × (*j*_1_ + *j*_2_). The probability of receiving all the source packets from the systematic phase, *j*_1_ = *K*, is (1 − *ϵ*)^*K*^. Once all the source packets are received, redundant packets are not needed. If only some of the original packets are received, *j*_1_ < *K*, then at least *j*_2_ ≥ *K* − *j*_1_ packets are required from the nonsystematic phase for successful decoding. The probability of matrix **G** **H** having full rank *K* when *j*_1_ columns are independent is given by ∏_*j*_3_=0_^*K*−*j*_1_−1^(1 − *q* ^*j*_3_−*j*_2_^) using the model in [44]. Combining both cases, *j*_1_ = *K* and *j*_1_ < *K*, we have *P*_*e*_ given in (10). Once we have the probability of successful decoding of all the *K* packets, the residual erasure rate is simply given by (11).

**Figure 4:**
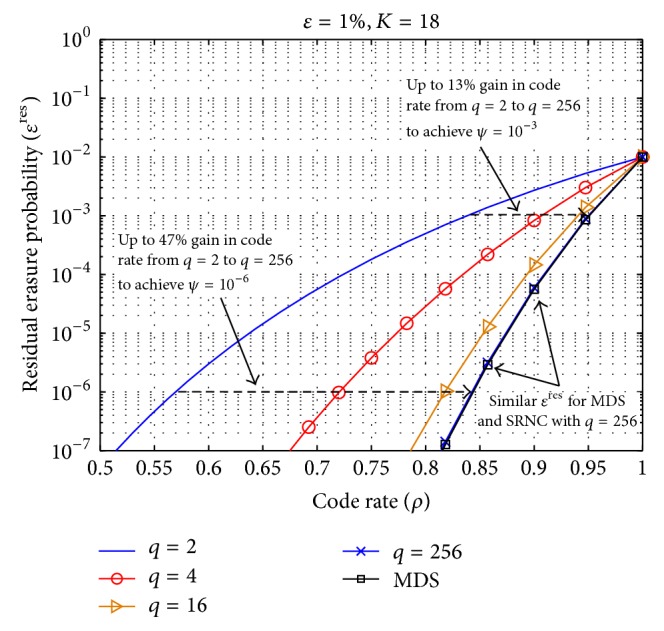
Effect of the field size on residual erasure probability.

In Figure 4, we present numerical results illustrating the effect of field size on *ϵ* ^res^. For our results, we choose *r*_APP_ = 200 kbps, *l* = 1400 bytes, and *T*_GoP_ = 1 second (*r*_APP_, *l*, and *T*_GoP_ are chosen corresponding to the realistic scenarios which we are considering for experiments in Section 7) such that *K* = ⌈(*r*_APP_ × *T*_GoP_)/*l*⌉ = 18. We vary the code rate *ρ* from 0.5 to 1 and consider erasure rate *ϵ* = 1%. Our results show that (i) a field size of *q* = 256 is enough for SRNC to guarantee performance similar to that of MDS codes and (ii) there is up to 47% gain in code rate, achievable with *q* = 256 as compared to *q* = 2 for a target erasure rate of *ψ* = 10^−6^. A higher code rate will result in greater budget allocation to the application rate which improves the QoE in the space domain.

## 6. Integration of Perceptual Semantics

We propose the use perceptual semantics to enhance specific perceptual features, following the model introduced in Section 3.2. Further, we integrate perceptual semantics with the joint time and space optimizations.

### 6.1. Formulation

We focus on using our proposed perceptual semantics for enhancement at source coding level. In single or scalable layer state-of-the-art video encoding, there are three types of resolution:defined-temporal (frame rate), amplitude (quantization step), and spatial (frame size).

We map enhancement of temporal features to higher frame rates and predominance of spatial features to higher spatial and amplitude frame resolution. In this way, dynamics of a scene can be more closely followed (temporal preference) and details of a scene can be better identified (spatial preference). The mapping is intuitive and nonintrusive and relies on the intrinsic architecture of video codecs currently in use to facilitate the video communications.

We propose mapping perceptual semantics to a system quantified with the variable *α* ∈ [0,1]. *α* = 0 and *α* = 1 express full preference of the spatial and temporal perceptual features, respectively. Intermediate values of *α* represent weighed spatiotemporal preferences.

We denote the feasible set of finite values of frame rate, as *F*_*T*_(*r*_APP_), while *F*_*S*_(*r*_APP_) is the feasible set for the spatial factors. Both are a function of the application layer rate *r*_APP_. Note that higher frame rates and frame sizes are possible to attain with higher *r*_APP_ [45]; hence, the feasible sets *F*_*S*_(*r*_APP_) and *F*_*T*_(*r*_APP_) corresponding to higher values of *r*_APP_ will contain a greater number of possible values that can be chosen from. For example, in the case of scalable video coding, if temporal dyadic scalability is performed, the available values of frame rate contained in *F*_*T*_(*r*_APP_) would be the base layer frame rate and the frame rates from the enhancement layers. The combination of all layers adds up to the full frame rate, that is, a full 30 Hz frame rate if *r*_APP_ is sufficient with *F*_*T*_(*r*_APP_) = {3.75 Hz, 7.5 Hz, 15 Hz, 30 Hz}.

In order to choose the appropriate value of frame rate and resolution according to our mapping of perceptual semantics, we formulate the following optimization function:

(12) rfr∗,sfr∗=max⁡ αr¯fr+1−αs¯frs.t. rfr∈FTrAPP, sfr∈FSrAPP,

where ${\bar{r}}_{fr}=r_{fr}/r_{fr}^{max}$ and ${\bar{s}}_{fr}=s_{fr}/s_{fr}^{max}$ are the normalized values of frame rate *r*_fr_ and spatial/amplitude resolution *s*_fr_ with respect to maximum available values set for the application.

Note that the optimization in (12) can be applied to single or scalable layer video coding.

### 6.2. Implementation

The implementation into the cross-layer optimization model from Section 3.4 is as follows.

The video streaming application uses a state-of-the-art codec such that the frame rate, frame size, and codec rate can be configured on-the-fly. In order to facilitate the role of perceptual semantics, we use a return path to send the tags chosen by the user. A semantics-aware adaptation block at the sender interprets the semantic tags coming from the end-user by mapping it to the proper decisions in (12) and forwarding to the video codec.

We propose the use of semantic web protocols to enable the semantic feedback to the transmitter through the APP-to-APP cross talk of the semantic tagging [46]. At the transport layer, the application-specific information can be encapsulated into RTCP feedback packets compliant with the extended reports defined in RFC4585. This way, the perceptual semantics feedback loop is coherent with the cross-layer optimization.

Our framework complies with the notion of a semantic information-based network. Hence, it is coherent with the content-aware trends in networking where the focus is on the network as a platform for information dissemination rather than simply an enabler of communication links. This framework can be mapped to information-centric networking (ICN) architectures such as publish/subscribe for live video as in [47]. A feasible topology mapping of ICN to our scenario is discussed in [48].

## 7. Experimental Results

### 7.1. Experimental Setup

The setup consists of a point-to-point streaming connection. The receiver and sender applications are connected through an emulated network using the NetEM emulator.

#### 7.1.1. Setup

Following Figure 3(b), we describe each block.

At application layer we use the state-of-the-art video codec VP8 [42]. At transport layer, we use the RTP/UDP protocol and a standard implementation of RTCP protocol for feedback. At network layer, each transport layer packet is encapsulated into an IP packet.

The online optimization has been implemented to output a rate control update of *R* ^*∗*^ with every new RTCP report, according to (7). The offline optimization uses a look-up table to output the optimal *r*_APP_^*∗*^ and code rate *ρ* ^*∗*^ values from the budget rate ${\tilde{r}}_{av}$.

We simulate SRNC coding by adding, for each GoP coming from the transport layer at rate *r*_APP_, redundant (dummy) packets such that *R* ^*∗*^ = *r*_APP_/*ρ* ^*∗*^.

#### 7.1.2. Network Emulation

With respect to erasures, packets are erased at the random rate *ϵ* when no erasure protection is performed. When SRNC is used, packets are erased corresponding to the residual erasure probability of SRNC $\epsilon =\epsilon_{res}(\epsilon ,q,r_{App}^{*},{\tilde{r}}_{av})$.

Congestion events are emulated as a drop (step-like) from maximum available rate *r*_av_^max^, which occurs halfway through one streaming session, at *T*/2. In practice, we use traffic shaping in the NetEm emulator to create the drops in *r*_av_^max^, such that *r*_av_ = *η* · *r*_av_^max^.

#### 7.1.3. Perceptual Semantics

The evaluation of the perceptual semantics approach of Section 6, integrated into the cross-layer optimization model, is performed using a simulation platform where the video streaming application is simulated by generating packets of size *l* encoded at a rate *r*_APP_. Network simulation follows the same guidelines as used with the time and space optimizations. All parameters in Table 2 apply, except for those related to the application layer.

We model a user's semantic tagging from temporal/spatial features with the parameter *α*. *α* may vary over time throughout one single streaming session, such that the sender is receiving feedback of these changes and adapts to them using (12). We assume that these tags are changed by the user every 10 s. We consider time variation of semantic tagging TAG_*TS*_ as a user alternating between spatial and temporal tags, each lasting 10 seconds.

Table 1 summarizes the feasible sets for values of frame rate *r*_fr_ dependent on *r*_APP_, in order to solve the algorithm in (12). The values chosen correspond to typical feasible combinations in current state-of-the-art codecs.

#### 7.1.4. Experiments

Table 2 summarizes the values of the parameters used for the experiments.

Experiments with and without space and time domain QoE optimizations are considered. Each experiment consists of one streaming session lasting *T* seconds. A large value of *T* (3 minutes) helps guarantee statistical significance with respect to erasure rates as well as spatiotemporal variations in the video.

For each experiment, a looped standard video sequence served as the input source. Furthermore, each experiment utilizes a specific value of *ϵ* and *η*. The ranges of values for *ϵ* are 0%–15%, while, for *η*, the range is from 100% to 50%.

The range of values considered for *r*_av_ and *r*_APP_ corresponds to realistic values for an application using a mobile satellite service, such as the BGAN network. Such network offers roughly maximum *r*_av_^max^ = 500 kbps in a best effort configuration. The propagation delay *τ*_*D*_ corresponding to a GEO-stationary satellite network is also configured in NetEm. The value of *ψ* was chosen according to 3GPP (3rd Generation Partnership Project) specifications for real-time scenarios.

### 7.2. Performance Metrics

#### 7.2.1. Spatiotemporal Perceptual Features of QoE

We measure the spatial and temporal perceptual features of QoE, coherent with the framework described in Section 3.1.2 and our spatiotemporal abstraction of the video.

Application layer information, both at the media and the bitstream levels, is collected at the receiver and the sender for offline performance assessment. A frame concealment strategy is used to avoid misalignment of sent/received video and the associated impact on full reference (FR) spatiotemporal video assessment. This implies that a lost frame is replaced in the sequence by the last frame received, using bitstream level data from actual frames sent and received. In addition, bitstream level data also provides frame play-out timestamps.

QoE_*ST*_ measures the spatiotemporal perceptual features of video. It is an FR media level metric, considered in [49], where it was shown to exhibit good correlation to subjective metrics. It is defined as QoE_*ST*_ = *μ*(*θ*) − *w* · *σ*(*θ*), with variables *θ*, *μ*(·), *σ*(·), and *ω* as follows. *θ* is, in our case, the vector with frame-by-frame full reference video quality metric SSIM from each experiment [50]. *μ*(·) indicates the mean value function. *σ*(·) is the standard deviation and *w* > 0 is a weight value. This metric considers the variability of quality throughout the streaming session; hence it is able to represent the impact of time variations in the network.

QoE_*T*_ measures the temporal perceptual features of video. It is a nonreference metric at bitstream level, representing video flow continuity. QoE_*T*_ is defined as the probability that no freezes appear in the video playback. Freezes are defined as events in which the time Δ elapsing between two consecutive frames displayed during video playback exceeds a tolerated threshold *ξ*. Hence we can define QoE_*T*_ as QoE_*T*_ = *P*{Δ < *ξ*}.

#### 7.2.2. Perceptual Semantics

We define the combined metric *Ω* to measure tradeoffs of using perceptual semantics with and without cross-layer optimization. It is defined as follows:

(13) $$\begin{array}{c} \Omega =w_{1}\cdot QoE_{A}+w_{2}\cdot QoE_{T}+w_{3}\cdot \left(\;1-\Delta_{\alpha }\;\right) \end{array}$$

with *w*_1_ + *w*_2_ + *w*_3_ = 1 and *Ω* ∈ [0,1]. $QoE_{A}=1-\bar{p}$, where $\bar{p}$ is the average packet loss rate at the receiver. $\Delta_{\alpha }=\left|\hat{\alpha }-\alpha \right|$ evaluates the performance of the perceptual semantics algorithm to determine whether the algorithm is achieving the user-demanded *α*. The best performance, that is, *Ω* = 1, occurs when no losses degrade the video (QoE_*A*_ → 1), freezes in playback are minimal (QoE_*T*_ → 1), and the perceptual semantic adaptation matches the one requested by the user (Δ_*α*_).

### 7.3. Joint Optimizations in the Time and Space Domains

The purpose of this experiment is to evaluate the performance of the joint optimizations in the time and space domains according to the proposed model in Figure 3(b). Hence, we consider degradations due to both congestions and erasures. Congestion events and erasures are emulated as in the previous sections with the parameters of Table 2 for Experiment (3). For each experiment a different value of *η* and *ϵ* was considered.

We compare the results of the joint optimization with a solution unaware of network dynamics, where the application layer is blind to the network dynamics, the transport layer is not performing any congestion control, and there is no protection against erasures.

#### 7.3.1. Effect on QoE_*T*_

**Figure 5:**
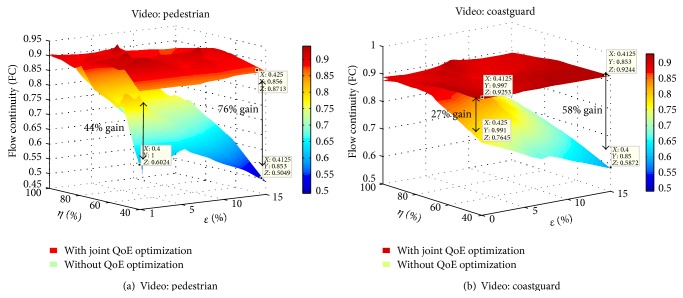
Flow continuity, QoE_*T*_ for joint QoE in space and time domain.

The three-dimensional QoE plots in Figure 5 show that, for all cases of congestion and erasures tested, the values of the flow continuity metric are all above 0.9 when using both optimizations. This implies that more than 90% of the time, the user is not experiencing freezes during video playback. The complete framework compared to a non-QoE optimized approach has gains of up to 60% in flow continuity.

#### 7.3.2. Effect on QoE_*ST*_

**Figure 6:**
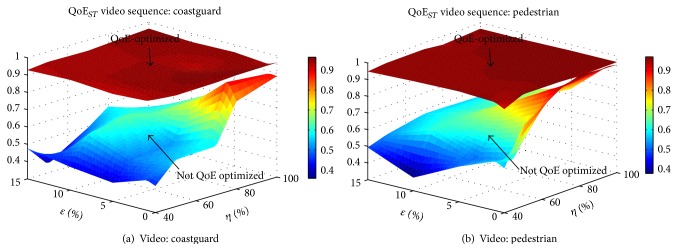
QoE_*ST*_ metrics for joint QoE in space and time domain.

The complete solution achieves in overall a planar surface in QoE_*ST*_, as shown in Figure 6. This implies that, regardless of both erasures and congestion affecting QoE, the combination of the online and offline strategies is able to deliver smooth performance.

Moreover, for all cases of congestion and erasures tested, the values of QoE_*ST*_ metric are all above 0.9 when using the complete QoE framework, guaranteeing very small variations in quality over time. The gain with respect to a nonoptimized approach is of up to 80%. The cases with higher improvement correspond to higher erasure rates and greater degree of congestion *η*. The gradient of the gains in QoE_*ST*_ for higher values of *η* is only dependent on the increase in erasure rates, while, for lower values of *η*, the gain increases jointly as *η* and *ϵ* increase.

These results represent a high QoE in the space domain together with smooth QoE performance throughout the entire streaming session, a characteristic highly valued by end-users. This behavior was observed with all video sequences tested.

### 7.4. Trade-Offs in the Decoupling Approach

We comment on the trade-offs by analyzing the isolated performance of both time and space optimizations and their effects on the metrics.

#### 7.4.1. Optimization in Time Domain

**Figure 7:**
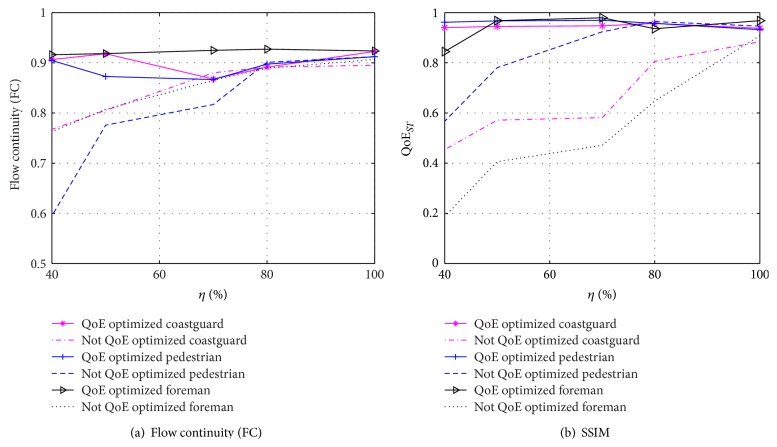
Evaluation of only QoE optimization in the time domain.

In this case we consider degradations due to congestion only and compare the performance to a solution that is unaware of such degradations. The results are summarized in Figure 7 for videos* coastguard, pedestrian,* and* foreman*. The parameters for this experiment correspond to Experiment (1) from Table 2.

*(a) Effect on QoE*_*T*_. As can be observed from Figure 7(a), the flow continuity measured with QoE_*T*_ is improved up to 50%. The highest advantage is achieved for lower values of *η*.

*(b) Effect on QoE*_*ST*_.The online optimization is able to avoid congestion events; hence packet loss due to congestion is minimized, proving that the space-time decoupling premise is valid. As a consequence, the improvements in QoE are not only in time domain metrics but also in the space domain.

It can be observed from Figure 7(b) that the most significant improvements occur for congestion events with *η* < 70% in QoE_*ST*_, with improvement of over 100%. For higher values of *η*, the metric also shows a gain from 4.5% to 50% using the QoE optimization in the time domain.

#### 7.4.2. Optimization in the Space Domain

In this experiment we compare the optimization in the space domain (where for each *r*_av_ an optimal *ρ* ^*∗*^ is obtained to configure and use SRNC) to a nonoptimized strategy with no erasure protection (*ρ* = 1). This case considers only degradations in the network due to erasures. We assume *r*_av_ is constant throughout the entire streaming session (*η* = 100%; there is no congestion). We assume the transmission rate *R* = *r*_av_, and ${\tilde{r}}_{av}=r_{av}$. For each experiment, there is a corresponding pair of values (*ϵ*, *r*_av_). The parameters are set as in Table 2 for Experiment (2).

*(a) Effect on QoE*_*T*_. By optimizing the rate budget ${\tilde{r}}_{av}$ when using SRNC, we ensure that the redundancy added will not congest the network. QoE in the time domain is therefore not affected by the use of SRNC, as is intended in our decoupling approach.

Notwithstanding, SRNC, similar to other block erasure codes, adds delay at encoding/decoding. The systematic characteristic of SRNC as well as the possibility of performing progressive RNC decoding significantly reduces the delays imposed by erasure protection. Therefore, we can assume a reduced start-up delay in the video playback. This small price to pay has a duration not longer than a GoP, *T*_GoP_, thereby guaranteeing minimal impact of SRNC on QoE in the time domain.

**Figure 8:**
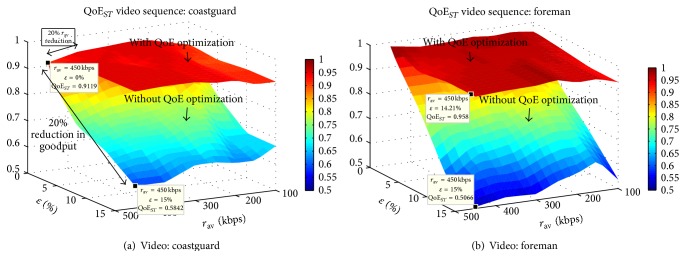
QoE optimization in the space domain. QoE_*ST*_ metric.

*(b) Effect on QoE*_*ST*_.  Figure 8 shows the results for videos* foreman* and* coastguard*, in our three-dimensional analysis of QoE, where we plot QoE metrics versus *ϵ* versus *η*. Using the optimization in the space domain, the main advantage is achieved in scenarios with high available rate and high erasure rates, with up to 38% improvement in QoE_*ST*_ metric compared to a nonoptimized strategy. The higher advantage occurs for higher values of *r*_av_.

We show minimal effects of the decoupling approach on QoE_*ST*_ with the following example. Consider the surface representing the case where space optimization is not utilized in Figure 8. In an erasureless scenario (*ϵ* = 0, *r*_av_ = *r*_APP_), reduction of 20% in *r*_APP_ has degradation of under 1% in QoE_*ST*_, while reduction in goodput due to erasures *ϵ* = 20% represents 40% degradation in QoE_*ST*_. This shows that the spatial perceptual features of QoE are not sacrificed when part of the budget rate is used for erasure protection. This is confirmed by the smooth performance of our solution in Figure 8. Due to the joint operating optimizations in both the time and space domains, we gain benefits in both. In time a rate ${\tilde{r}}_{av}$ that avoids congestion is ensured. In space we optimally assign the resources for SRNC (*ρ* ^*∗*^) and application layer (*r*_APP_^*∗*^) such that we obtain a target residual erasure rate *ψ* and the spatial perceptual features of QoE are preserved.

### 7.5. Perceptual Semantics with and without Time and Space Optimizations

To our knowledge, there is no similar framework in the literature to match our proposed perceptual semantics framework and hence comparison to solutions that do not have a similar goal would be unfair. Therefore, our results focus on not having such a kind of framework. In order to observe the combined effects of the adaptation through perceptual semantics with the cross-layer optimization, we compare the use of cross-layer optimization to cope with the network constraints to a situation where it is not used.

**Figure 9:**
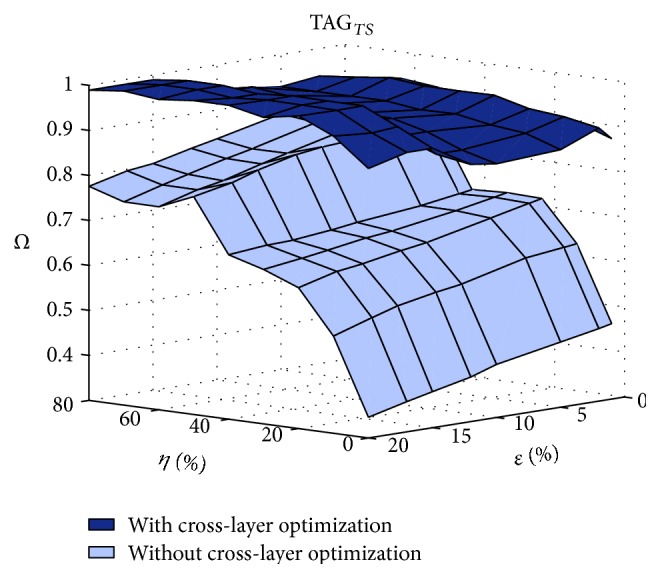
*Ω* for time-varying semantic tagging TAG_*TS*_.

We analyze the effects of time-varying perceptual tagging, representing a realistic case where the user identifies different situations that demand attention towards temporal or spatial features. These variations are represented as alternations of temporal and spatial tagging.  Figure 9 shows the performance in terms of the combined metric *Ω*.

In addition to achieving the expected *α* demanded through the use of semantic tagging, the performance is above 80% regardless of the degradations of the network, thanks to the cross-layer optimization. The performance is highly degraded due to congestion as well as erasures when no cross-layer optimization is used, with performance dropping to 40%.

Figure 9 confirms the analysis by showing that the cross-layer optimization preserves the perceptual semantics.

## 8. Conclusions

In this work, we proposed a solution to deliver point-to-point video services in best effort satellite networks for purposes beyond recreational, such as for situational awareness. We used QoE framework to decouple the problems inherent to the scenario, relating congestion with freezes in the time domain and packet erasures with artifacts in the space domain. Both impairments degrade the QoE of video and as a result the ability of video to help gain situational awareness. Our decoupled approach facilitates the design to optimize QoE both in the time and in the space domains, thereby providing a feasible solution for dynamic adaptive streaming tailored to the scenario's needs. As a consequence of decoupling and tackling these two problems separately, we have performed a time/space graphical analysis with varying network conditions in form of congestion and erasures. Furthermore, driven by the temporal-spatial abstraction of video and its perceptual features, we presented a novel model for perceptual semantics, based upon the user's demands. We also proposed the framework to be integrated into an interactive video adaptive solution, for user situational awareness. We discussed how to practically implement perceptual semantics into an adaptive loop that works with underlying cross-layer optimization. Our experimental results showed the benefits of this decoupled approach in terms of objective QoE metrics. We were able to achieve homogenous high performance, regardless of both erasures and congestion degrading the network. Our simulation results also showed how perceptual semantic tagging achieved the expected user demands while the underlying cross-layer optimization preserved performance. Future work includes the extension of our analysis to the general network where intermediate nodes can perform coding for higher reliability and throughput. Furthermore, other aspects of QoE, such as context, can be studied within our decoupled QoE framework. In addition, extensions of perceptual semantics in the ICN context will be pursued. The ICN umbrella allows the future consideration of our scheme for live multicast dissemination to a number of users assessing simultaneously an ongoing critical mission. The last mile networking elements in ICN could be in charge of the multicast distribution. Moreover, we will study more pertinent QoE metrics to match user's satisfaction when using perceptual semantics.

## Figures and Tables

**Table 1:** Feasible sets considered for simulation of perceptual semantics.

*r*_APP_ (in kbps)	Feasible set *F*_*T*_	Feasible set *F*_*S*_
*r*_APP_ ≤ 64	{3.75,7.5,10,15}	{QCIF}
64 < *r*_APP_ ≤ 192	{3.75,7.5,10,15}	{QCIF, CIF}
192 < *r*_APP_ ≤ 384	{3.75,7.5,10,15}	{CIF, QCIF}
384 < *r*_APP_ ≤ 500	{3.75,7.5,10,15,30}	{QCIF, CIF, 640 × 360}

**Table 2:** Parameters in experimental setup for time and space optimizations.

Experiments		(1) QoE (time)	(2) QoE (space)	(3) Joint QoE

Video sequences		*Pedestrian, foreman, *and* coastguard *		
*T* (streaming time)		3 min		

APP	*N*_frame_	15		
	*r*_fr_	15 fps		
	*r*_APP_(*t*_1_)	500 kbps	[100–500 kbps]	500 kbps

Transport	pkt size *l*	1400 B		
	*T*_samp_	2 s		

Network	*q*	—	256	256
	ψ	—	10^−3^ [38]	10^−3^ [38]

Network emulation	*τ*_*D*_	250 ms		
	ϵ	no	[0–15] %	[0–15] %
	*r*_av_^max^	500 kbps	[100–500 kbps]	500 kbps
	η	[100–50] %	0%	[100–50] %
